# Supplementary figures and images for: Crucial Role of ppGpp in the Resilience of Escherichia coli to Growth Disruption
Source: mSphere. 2020 Dec 23;5(6):e01132-20. doi: 10.1128/mSphere.01132-20 (PMC7763551; doi:10.1128/mSphere.01132-20)

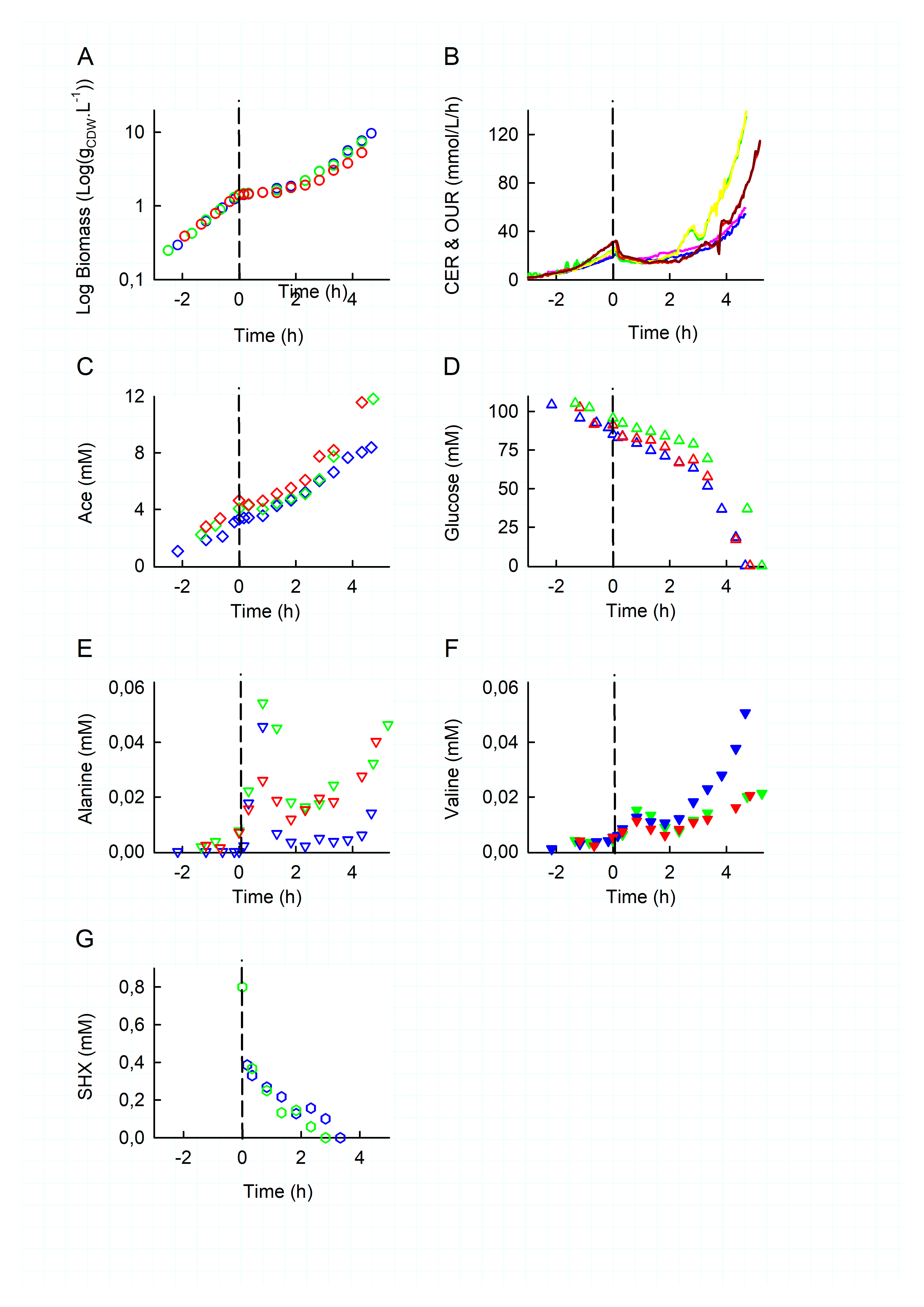

Supplement: FIG S1 [file mSphere.01132-20-sf001.tif]

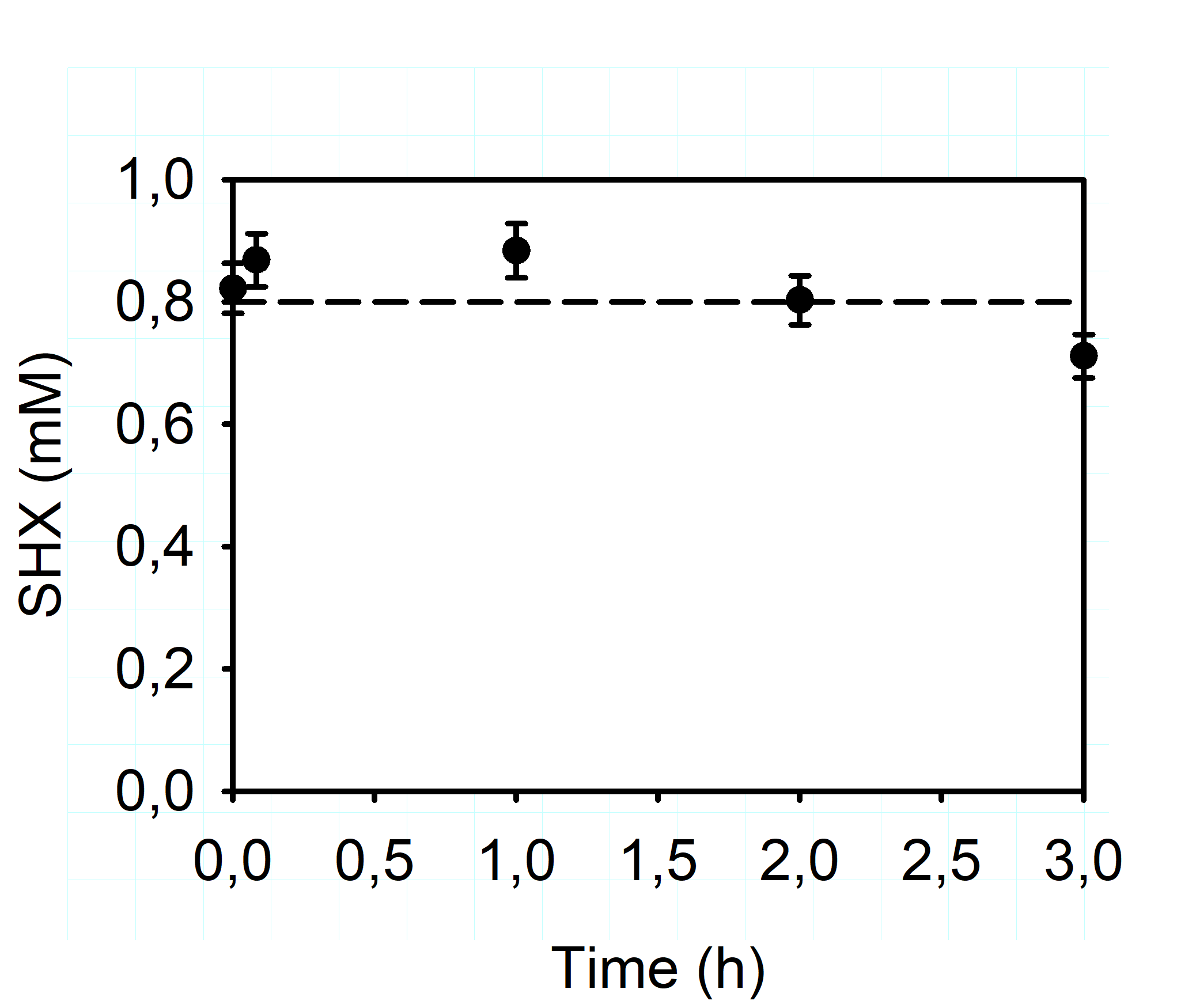

Supplement: FIG S2 [file mSphere.01132-20-sf002.tif]

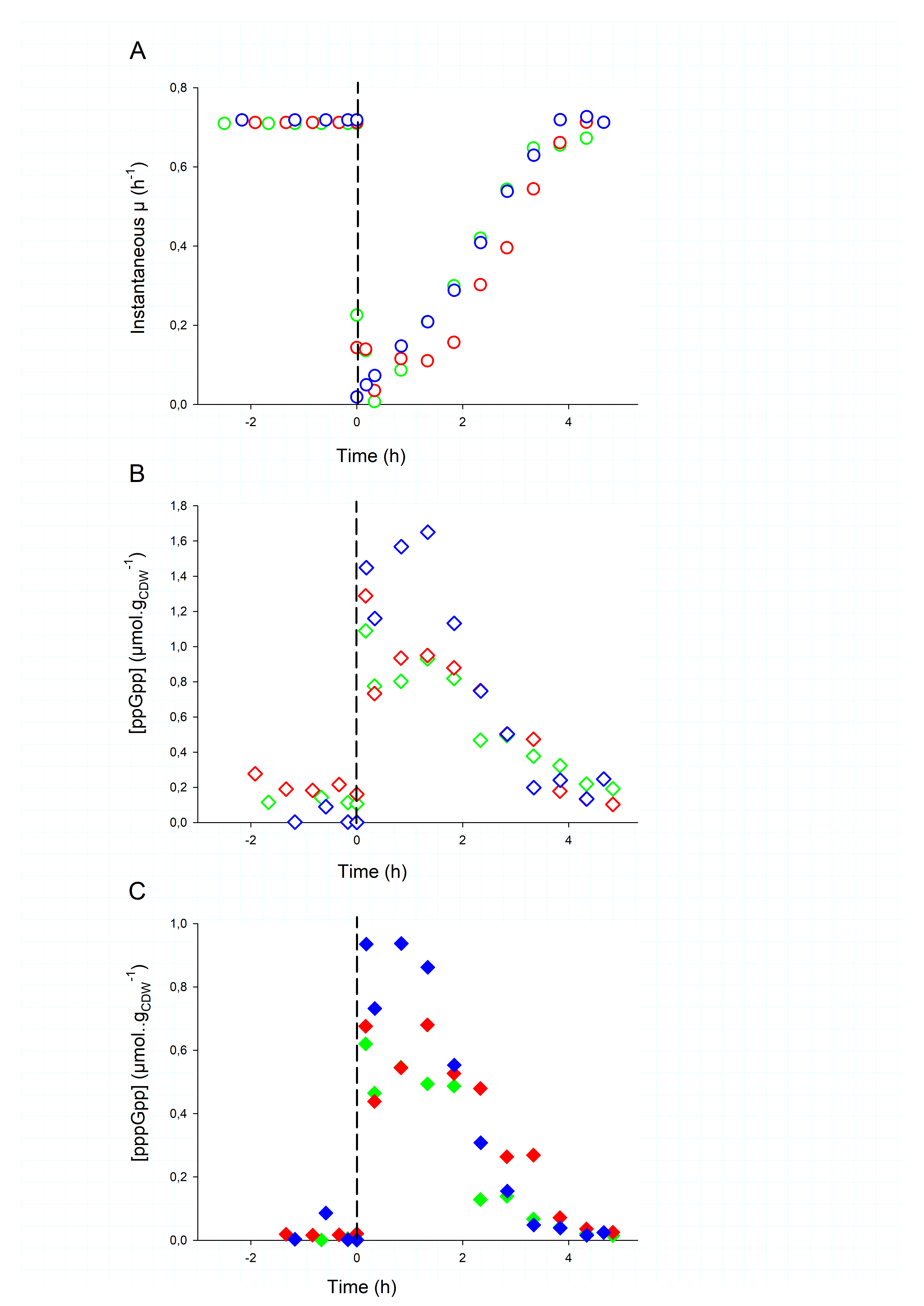

Supplement: FIG S3 [file mSphere.01132-20-sf003.tif]

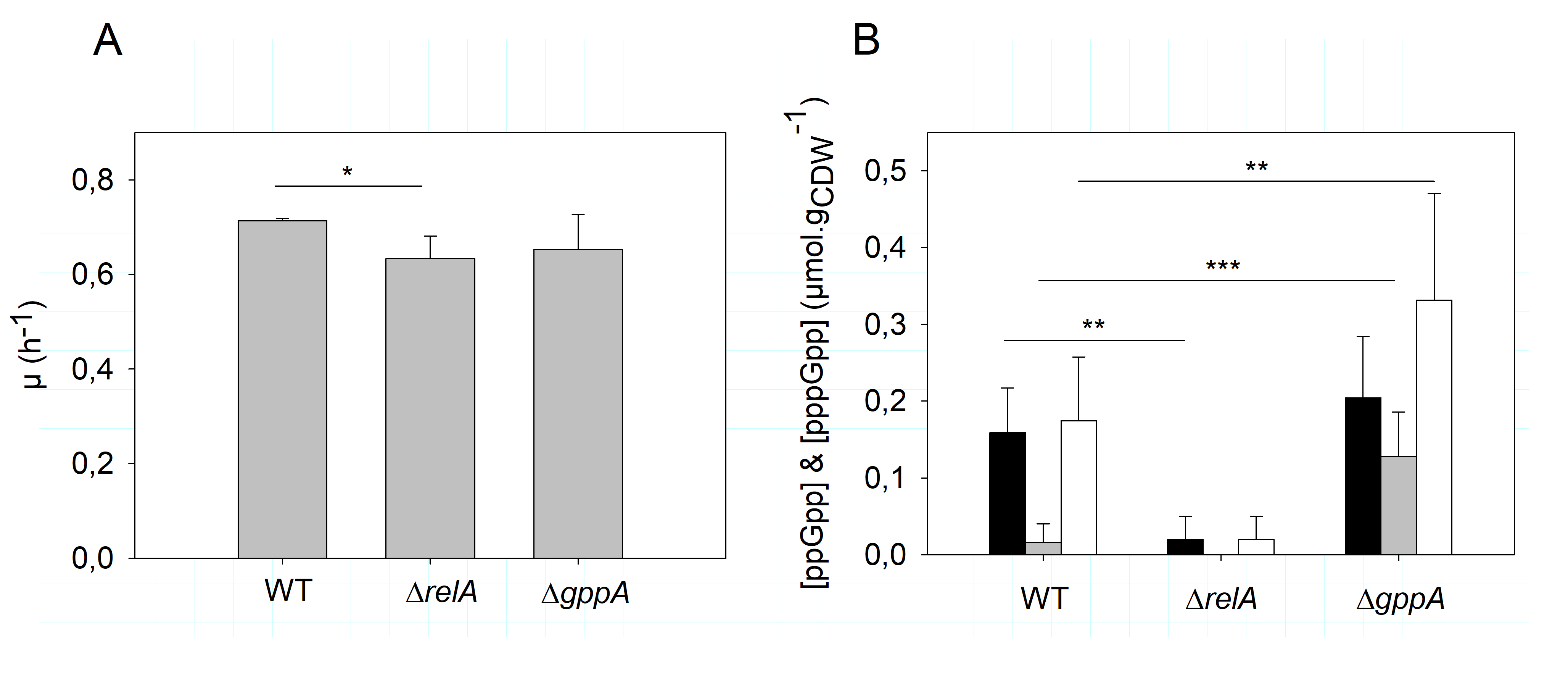

Supplement: FIG S4 [file mSphere.01132-20-sf004.tif]

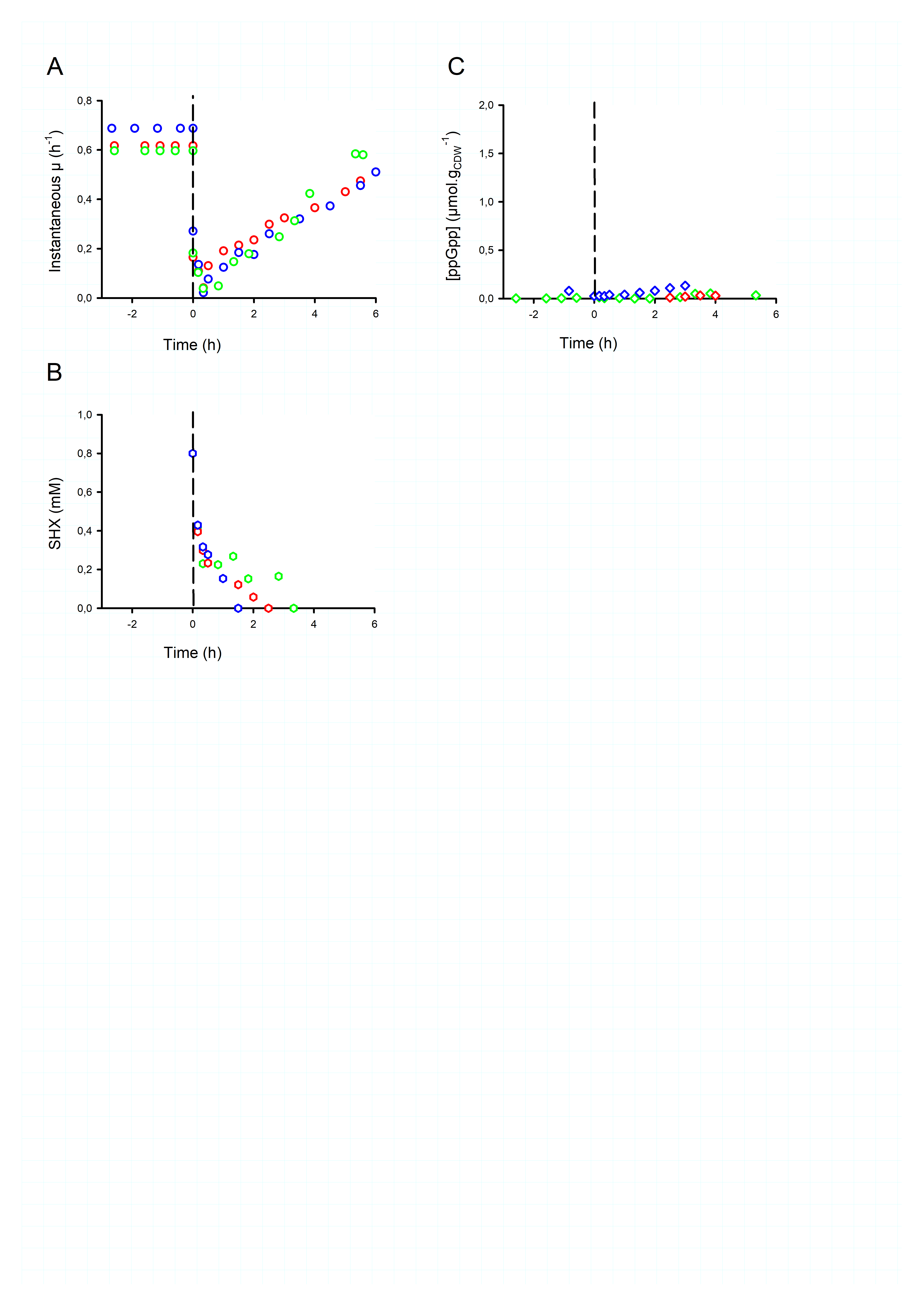

Supplement: FIG S5 [file mSphere.01132-20-sf005.tif]

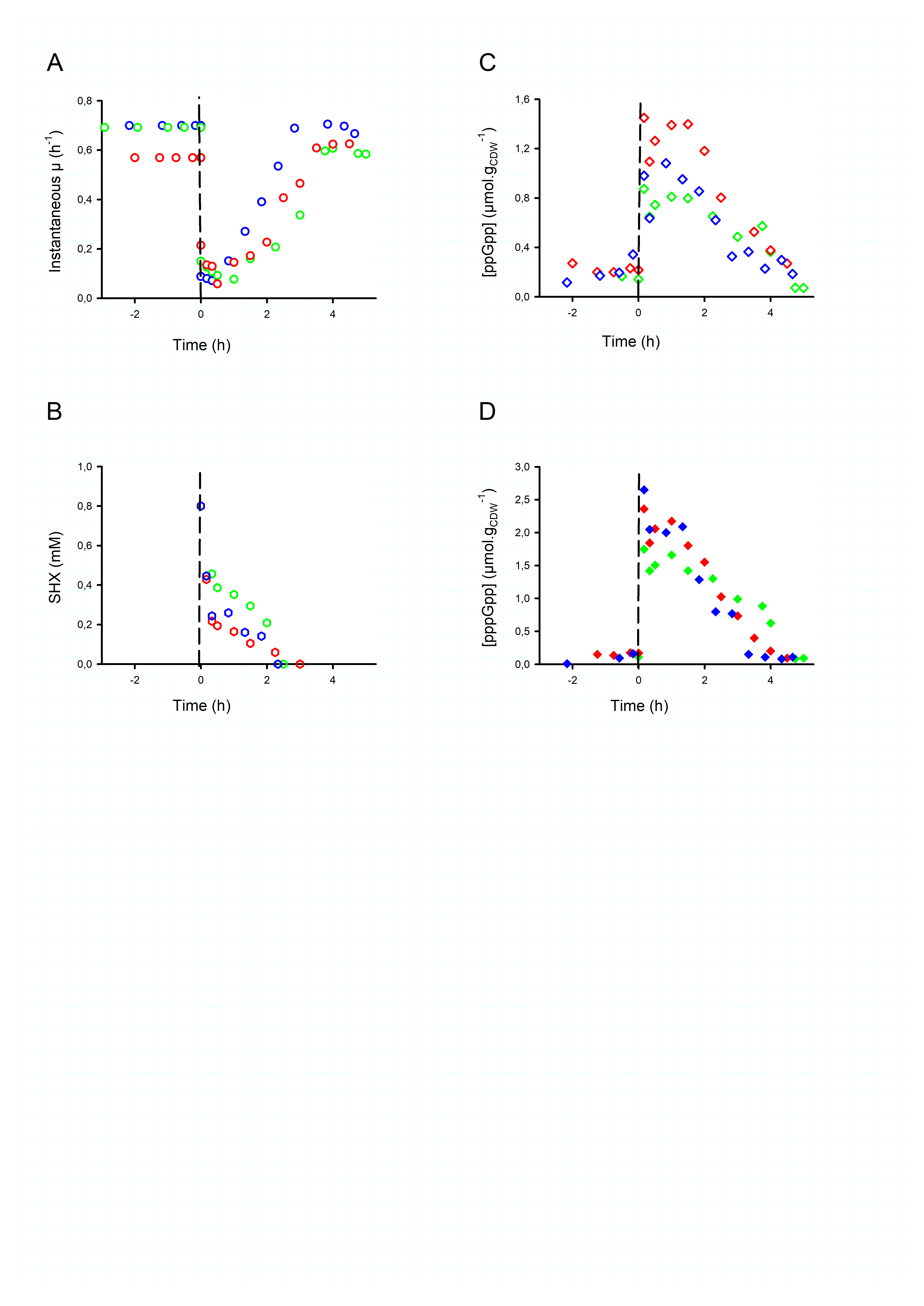

Supplement: FIG S6 [file mSphere.01132-20-sf006.tif]
