# Supplementary material for: Crucial Role of ppGpp in the Resilience of Escherichia coli to Growth Disruption
Source: mSphere. 2020 Dec 23;5(6):e01132-20. doi: 10.1128/mSphere.01132-20 (PMC7763551; doi:10.1128/mSphere.01132-20)
Supplement: TABLE S1 [file mSphere.01132-20-st001.docx]

**Table S1:** Fluxes of alanine and valine excretion following SHX addition

|  | Alanine | | | Valine | | |
| --- | --- | --- | --- | --- | --- | --- |
|  | J_AE_^a^  µmol·(g_CDW_·h)^−1^ | J_AX_^b^  µmol·(g_CDW_·h)^−1^ | %  (J_AE_/J_AX_) | J_VE_^a^  µmol·(g_CDW_·h)^−1^ | J_VX_^b^  µmol·(g_CDW_·h)^−1^ | %  (J_VE_/J_VX_) |
| WT | 32.2 ± 14.5 | 348.3 ± 2.5 | 9.2 ± 4.2 | 6.8 ± 2.2 | 286.7 ± 2.1 | 2.4 ± 0.8 |
| Δ*relA* | 73.7 ± 8.0 | 309.0 ± 23.1 | 23.9 ± 3.1 | 5.5 ± 1.0 | 254.7 ± 18.9 | 2.1 ± 0.4 |
| Δ*gppA* | 31.7 ± 22.1 | 332.3 ± 51.7 | 9.5 ± 6.8 | 3.2 ± 1.3 | 262.7 ± 29.2 | 1.2 ± 0.5 |

^a^ Maximal excretion flux of alanine or valine calculated during the first hour after SHX addition.

^b^Anabolic flux of alanine or valine into biomass inferred from (1) genome-scale metabolic model of *E. coli* K-12 MG1655 and the growth rate measured before SHX addition.

Reference:

1. Feist AM, Herrgård MJ, Thiele I, Reed JL, Palsson BØ. 2009. Reconstruction of biochemical networks in microorganisms. Nat Rev Microbiol 7:129–143.
