# Supplementary material for: Crucial Role of ppGpp in the Resilience of Escherichia coli to Growth Disruption
Source: mSphere. 2020 Dec 23;5(6):e01132-20. doi: 10.1128/mSphere.01132-20 (PMC7763551; doi:10.1128/mSphere.01132-20)
Supplement: TABLE S2 [file mSphere.01132-20-st002.docx]

**Table S2:** Strains, plasmids and primers used in this study

| **Bacterial strains, plasmids, and primers** | **Description (genotype and/or relevant characteristics) or sequence of oligonucleotide primers** | **Reference or source** |
| --- | --- | --- |
| **Strains** |  |  |
| *E. coli* K-12 MG1655 WT | F^–^ λ^–^ *ilvG*^–^ *rfb-50* *rph-1* | This study |
| *E. coli* K-12 MG1655 Δ*relA* | *E. coli* K-12 MG1655 Δ*relA* | This study |
| *E. coli* K-12 MG1655 Δ*gppA* | *E. coli* K-12 MG1655 Δ*gppA* | This study |
| BW25113 WT | Δ(araD-araB)567 Δ(rhaD-rhaB)568 ΔlacZ4787 (::rrnB-3) hsdR514 rph-1 | (1) |
| BW25113 *ΔrelA::Kan* | BW25113 *ΔrelA::Kan* | (1) |
| BW25113 *ΔgppA::Kan* | BW25113 *ΔgppA::Kan* | (1) |
| **Plasmids** |  |  |
| pCP20 | Flp recombinase gene, FLP, chloramphenicol and ampicillin resistant genes, and temperature sensitive replication | (2) |
| **Primers** |  |  |
| RelA_Forward | 5'-GATTTGCCGATTTCGGCAGGTCTG | This study |
| RelA_Reverse | 5'-ACTGTGGATCATAACCCTTTCCTC | This study |
| GppA_Forward | 5'- AGAATGCAGCCAACACAGAGACA | This study |
| GppA_Reverse | 5'- TCAAGGCCGGAAAAGATGCGT | This study |

References:

1. Baba T, Ara T, Hasegawa M, Takai Y, Okumura Y, Baba M, Datsenko KA, Tomita M, Wanner BL, Mori H. 2006. Construction of Escherichia coli K-12 in-frame, single-gene knockout mutants: the Keio collection. Mol Syst Biol 2:2006.0008.

2. Cherepanov PP, Wackernagel W. 1995. Gene disruption in Escherichia coli: TcR and KmR cassettes with the option of Flp-catalyzed excision of the antibiotic-resistance determinant. Gene 158:9–14.
